# Total severity score and age predict long-term hospitalization in COVID-19 pneumonia

**DOI:** 10.3389/fmed.2023.1103701

**Published:** 2023-04-12

**Authors:** Athina Nasoufidou, Marianthi Kavelidou, Theodora Griva, Eleni Melikidou, Charalampos Maskalidis, Konstantina Machaira, Barbara Nikolaidou

**Affiliations:** ^1^Internal Medicine Department, General Hospital Agios Pavlos, Thessaloniki, Greece; ^2^Surgery Department, General Hospital Agios Pavlos, Thessaloniki, Greece; ^3^Radiology Department, General Hospital Agios Pavlos, Thessaloniki, Greece

**Keywords:** COVID-19, total severity score, pneumonia, hospitalization length, chest computed tomography

## Abstract

**Background:**

Severe COVID-19 pneumonia implies increased oxygen demands and length of hospitalization (LOS). We aimed to assess a possible correlation between LOS and COVID-19 patients' clinical laboratory data of admission, including the total severity score (TSS) from chest computed tomography (CT).

**Methods:**

Data were assessed retrospectively at the General Hospital “Agios Pavlos” in Greece. Clinical laboratory data, TSS, and LOS were recorded.

**Results:**

A total of 317 patients, 136 women and 181 men, with a mean age of 66.58 ± 16.02 years were studied. Significant comorbidities were hypertension (56.5%), dyslipidemia (33.8%), type 2 diabetes mellitus (22.7%), coronary heart disease (12.9%), underlying pulmonary disease (10.1%), and malignancy (4.4%). Inpatient time was related to age (*p* < 0.001), TSS (*p* < 0.001), time from symptom onset to hospitalization (*p* = 0.006), inhaled oxygen fraction (*p* < 0.001), fibrinogen (*p* = 0.024), d-dimers (*p* < 0.001), and C-reactive protein (*p* = 0.025), as well as a history of hypertension (*p* < 0.001) and type 2 diabetes mellitus (*p* < 0.008). The multivariate analysis showed a significant association of the LOS with age (*p* < 0.001) and TSS (*p* < 0.001) independent of the above-mentioned factors.

**Conclusion:**

Early identification of disease severity using the TSS and patients' age could be useful for inpatient resource allocation and for maintaining vigilance for those requiring long-term hospitalizations.

## Introduction

Novel coronavirus (2019-nCoV) is the cause of the ongoing pandemic, which emerged at the end of 2019. It has caused 6.5 million deaths globally according to data collected by the World Health Organization (WHO) (1). Grouped in the family of Coronaviridae, SARS-CoV-2 is an RNA virus (2) named after the acute respiratory syndrome that it causes. It primarily targets respiratory epithelial cells and leads to pneumonia (3). Progression to respiratory failure and acute respiratory distress syndrome (ARDS) depends on the immunological status of the affected individual and the severity of the inflammatory response (2–5). Hyperactivation of immunological response and subsequent release of excessive amounts of pro-inflammatory cytokines induce a major lung injury (3). Clinical manifestations of severe disease including dyspnea, oxygen desaturation, elevated respiratory rate or a PaO2/FiO2 rate of < 300 (3, 6), ARDS, and septic shock indicate critical illness and lead to multiorgan failure, intensive care unit (ICU) hospitalization and death (6).

The diagnostic approach relies on the reverse transcriptase–polymerase chain reaction (RT-PCR) assay or chest computed tomography (CT), or both (7). RT-PCR is widely used for detecting the COVID-19 virus in respiratory specimens (7, 8). It is considered the gold standard method; however, reagents and personnel are required to perform RT-PCR, and several hours to obtain the result. The poor quality of viral messenger RNA can also lead to false-negative results even in symptomatic individuals (8, 9). For such cases in which the rate of suspicion is high, it is suggested that chest CT can be used as a tool to screen and confirm the diagnosis (10).

Imaging assessment of pneumonia with chest CT has been commonly used to reveal pulmonary involvement (11–13). In the case of COVID-19, ground-glass opacity distributed in the peripheral lung fields, with or without consolidation, is the most common feature. Other findings include the reticular or interlobular thickening pattern known as “crazy paving” (9, 14), bronchial wall thickening, pleural effusions, and hilar lymphadenopathy (14, 15). None of these CT findings specifically highlights COVID-19 pneumonia since they can also demonstrate other viral cases of pneumonia or even interstitial lung disease (16, 17). In an attempt to quantify the CT findings, Wang et al. suggested dividing the lungs into three zones and estimating the percentage of damage. Graded from 0 to 4 (where 0 = 0% involvement, 1 = < 25% involvement, 2 = 25% to < 50% involvement, 3 = 50% to < 75% involvement, and 4 = 75% or greater involvement), the maximum possible score is 24, which is the summation of the six scores for each zone (18). This score reflects the severity of the lung disease.

In this study, we aimed to investigate how the clinical findings, laboratory values, and total severity score (TSS) in chest CT can be associated with the length of hospital stay (LOS). Furthermore, we aimed to determine the impact of these factors on the LOS.

## Materials and methods

### Patients

We conducted a retrospective study of patients with a diagnosis of COVID-19 during a 4-month period from November 2020 to February 2021, who were admitted to a hospital designated for the admission of COVID-19 patients. All patients had a positive SARS-CoV-2 detection by quantitative RT-PCR of nasopharyngeal swabs. The study was approved by the institutional ethics board of the hospital, and informed consent was waived by the ethics committee for this retrospective study. The authors followed the ethical principles defined by the Declaration of Helsinki.

### Criteria of hospital admission

According to the recommendations of the Centers for Disease Control and Prevention (CDC) (19) and the National Public Health Organization (20), inpatient management is provided for severe and critical cases of the disease. Hospitalization is recommended for patients with pneumonia with a positive RT-PCR test who meet two or more of the following criteria: (1) a breathing rate >20 breaths/minute, (2) hypoxia defined as a SpO2 ≤ 94%, (3) lung involvement on chest imaging >50%, and (4) a PO2/FiO2 < 300. Critical illness, respiratory failure, shock, and multiorgan system dysfunction are also conditions that necessitate hospitalization.

### Data collection

Demographic, clinical, laboratory, and outcome data for each patient were extracted from electronic medical records using a standardized data collection form. Clinical data refer to symptoms and vital signs (blood pressure, pulse oximeter O2 saturation, heart rate, temperature, respiratory rate) on admission, time from symptom onset to hospital admission, and LOS. Special emphasis was placed on comorbidities recording hypertension, type 2 diabetes mellitus, coronary artery disease, heart failure, atrial fibrillation, pulmonary embolism, dyslipidemia, chronic obstructive pulmonary disease, peripheral artery disease, chronic kidney disease, history of stroke, malignancy, and anxiety, as possible modifiers of prolonged hospitalization and outcome.

Laboratory tests included complete blood count, kidney function, liver function, C-reactive protein, troponin, lactate dehydrogenase, ferritin, procalcitonin, erythrocyte sedimentation rate, prothrombin time, activated partial thromboplastin time, thrombin time, fibrinogen, D-dimer, and lipid profile. Additional information including electrocardiogram and arterial blood gas findings was obtained. Chest radiography and chest computed tomography were also performed within 24 h of admission, and the total severity score was recorded.

### Statistical analysis

Data analysis was performed using SPSS 27 (Statistical Package for Social Sciences 27, SPSS Inc., Chicago, IL, USA) software, version 26. According to the normality of the data distribution, frequencies were used for qualitative variables, and mean ± standard deviation (SD) or median (IQR, interquartile range) for continuous variables. Categorical data were expressed as percentages and compared by the chi-square test. Differences between groups were evaluated by using the *t*-test for parametric variables and the Mann–Whitney test for non-parametric variables. Correlations between continuous variables were performed with the parametric Pearson or the nonparametric Spearman's Rho correlation coefficient. To further investigate the factors associated with prolonged hospitalization, multivariate analysis with linear regression or multiple linear regression model was performed. A probability value of *p* ≤ 0.05 was considered statistically significant.

## Results

### Baseline information

**Figure 1:**
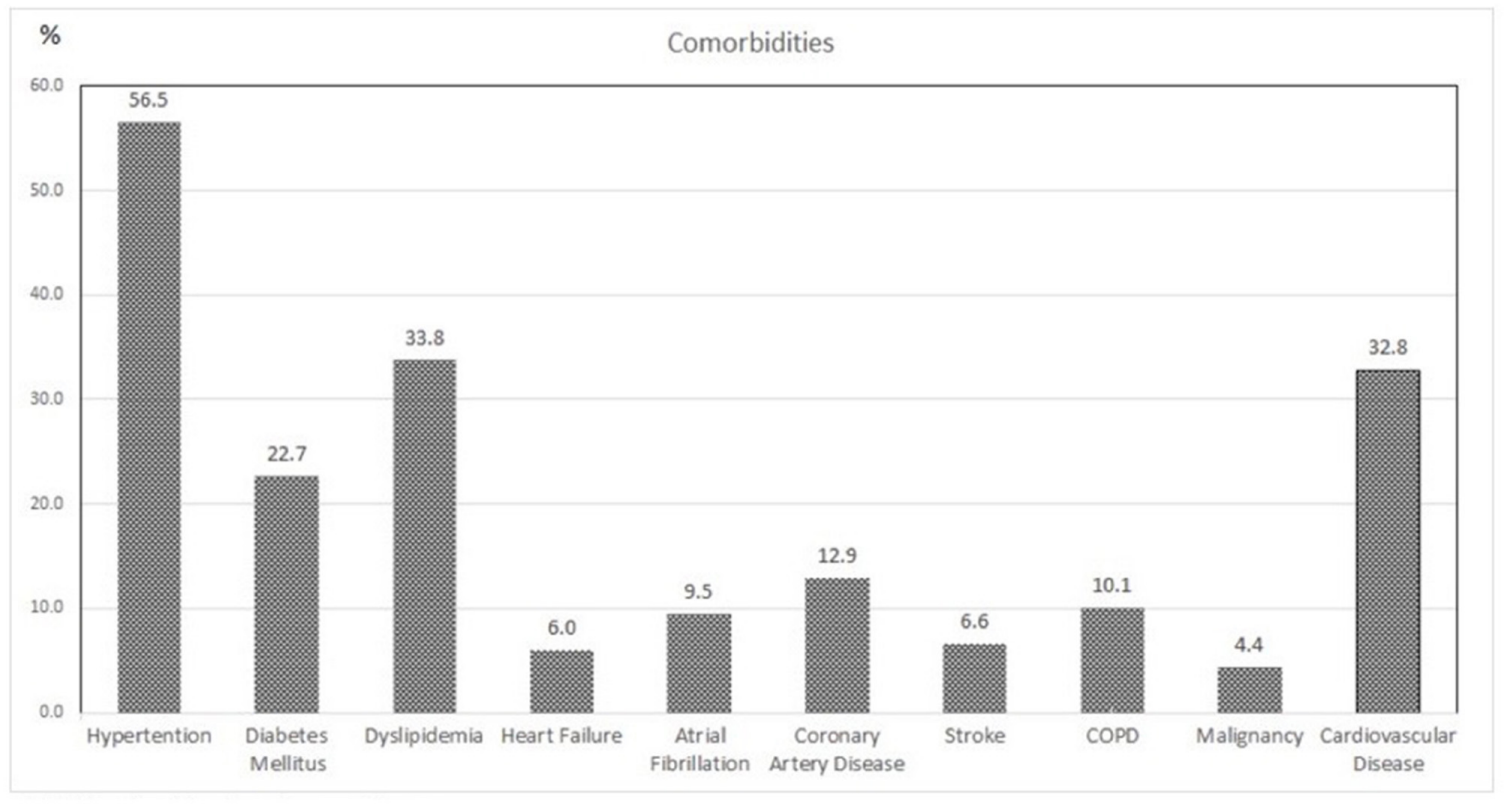
Associated comorbidities of the study population. COPD, chronic obstructive pulmonary disease.

A total of 317 patients with a median age of 66.58 ± 16.07 were included, and 57% of them were male patients. Baseline characteristics of the population are given in Table 1. The LOS was 16 ± 11.71 days, and the duration between symptom onset and admission was 6 ± 3.61 days. The TSS had a median value of 9 (IQR: 5) days, classifying the mean pulmonary involvement as mild to moderate (< 50%). Almost 27% of the patients had no underlying conditions, while the remaining approximately three-quarters (72.97%) of the patients had at least one comorbidity. Hypertension was present in 56.5% of the population, making it the most common comorbidity, followed by dyslipidemia, type 2 diabetes mellitus, and coronary artery disease (Table 1, Figure 1). The laboratory values showed a significant increase in the inflammatory blood markers, including C-reactive protein 3.5 mg/dl (IQR: 5.8), fibrinogen 506 mg/dl (IQR: 167), ferritin μg/L 738 (IQR: 1078), and D-dimer 0.46 mg/L (IQR: 0.49), reflecting the presence of an acute viral infection (Table 1).

**Table 1:** Characteristics of the study population.

**Variable**	**COVID-19 hospitalized patients (*n* = 317)**
Age (years)	66.58 ± 16.07
Males (%)	57
Days of hospitalization	16 ± 11.71
Days (from symptom onset to admission)	6 ± 3.61
Total severity score (TSS)	9 (5)
**Symptoms and signs on admission**	
Dyspnea (%)	30
Hypoxia (%)	15.5
SpO_2_	94.28 ± 8.25
FiO_2_	0.21 (0.14)
Systolic BP (mmHg)	134.75 ± 20.83
Heart rate (beats/min)	78 (21)
Temperature (°C)	36.6 (0.7)
**Laboratory tests on admission**	
WBC x10^9^/l	7 (3.83)
Hemoglobin g/dl	13.2 (2.2)
PLT /ml	251.000 (153.000)
Cre admission (mg/dl)	0.83 (0.29)
INR admission	1.05 (0.1)
aPTT admission (sec)	30.4 (4.80)
Fibrinogen admission (mg/dl)	506 (167)
D-dimer admission (mg/L)	0.46 (0.49)
CRP admission (mg/dl)	3.5 (5.8)
Ferritin μg/l	738 (1.078)
**Comorbidities**	
Hypertension (%)	56.5
Dyslipidemia (%)	33.8
Diabetes mellitus type 2 (%)	22.7
Coronary artery disease (%)	12.9
Heart failure (%)	6
Atrial fibrillation (%)	9.5
Stroke (%)	6.6
COPD (%)	10.1
Malignancy (%)	4.4
Cardiovascular disease (%)	32.8

aPTT, activated partial thromboplastin time; BP, blood pressure; COPD, chronic obstructive pulmonary disease; Cre, creatinine; CRP, C-reactive protein; FiO_2_, fraction of inspired oxygen; INR, international normalized ratio; PLT, platelets; SpO_2_, oxygen saturation; WBC, white blood cells.

Data are expressed as mean ± standard deviation (SD) or median (interquartile range).

### Association between factors of admission and LOS

From the demographic characteristics and clinical values of the studied population, LOS was associated with older age (*r* = 0.426, *p* < 0.001) and with time from symptom onset to hospital admission (*r* = −0.159, *p*: 0.006). Moreover, from the oxygenation panel of admission and imaging reports, FiO2 (*r* = 0,249, *p* < 0.001) and TSS (*r* = 0.234, *p* < 0.001) were positively correlated with LOS. Considering the laboratory values of admission, LOS had a statistical correlation only with D-dimer (*r* = 0.322, *p* < 0.001) and C-reactive protein (*r* = 0.126, *p* < 0.03) and a reverse correlation with fibrinogen (*r* = −0.134, *p*: 0.024).

Diabetes mellitus type 2 (*r* = 0.153, *p*: 0.008) and hypertension (*r* = 0.291, *p* < 0.001) were the two factors that correlated with LOS. To determine the overall contribution of cardiovascular comorbidities to the outcome, the studied population was stratified into two groups: One group referred to those with a cardiovascular condition, including hypertension, type 2 diabetes mellitus, coronary artery disease, heart failure, atrial fibrillation, pulmonary embolism, dyslipidemia, peripheral artery disease, chronic kidney disease, and history of stroke, and the other group included those with chronic obstructive pulmonary disease, malignancy, and anxiety, grouping the patients with non-cardiovascular disorders. The presence of cardiovascular disease (*r* = −0,314, *p* < 0.001) was correlated with LOS.

In the multivariate analysis, the age (beta = 0.306, *p* < 0.001) and total severity score on admission (beta = 0.25, *p* < 0.001) were identified as independent predictors of the LOS in the study population, independent of the other factors (Table 2).

**Table 2:** Multiple linear regression model in the total population for days of hospitalization.

**Variable**	**Beta**	***p***
Age	0.306	< 0.001
Days (from symptom onset to admission)	0.047	0.483
FiO_2_ admission	0.006	0.927
Total severity score (TSS)	0.250	< 0.001
Diabetes mellitus type 2	0.045	0.495
Hypertension	0.017	0.837
Fibrinogen admission	0.064	0.315
D-dimer admission	0.028	0.663
CRP admission	0.050	0.434

Depending variable: days of hospitalization.

CRP, C-reactive protein; FiO_2_, fraction of inspired oxygen.

## Discussion

The COVID-19 pandemic has profoundly affected health systems worldwide. One of the many factors that have been partly responsible for the inability of healthcare institutions to cope with the pandemic was the prolonged hospital stay of the affected individuals. Many factors contributing to long-term hospitalizations have not been sufficiently studied. In this study, we assessed the main factors of admission that could possibly contribute to LOS in patients with COVID-19 pneumonia. A data analysis showed that older individuals and/or individuals with extended imaging pulmonary involvement on admission require considerable inpatient time. These two factors seem to be directly and positively associated with the LOS.

Focusing primarily on age and imaging reports, according to our results, we can distinguish patients with potentially substantial risk for a long-term hospital stay. This could be useful in the decision process of resource allocation in terms of healthcare services. Furthermore, a high rate of suspicion warrants intensified treatment and close surveillance, aiming to reduce adverse outcomes and complications. Emphasizing on age, elderly patients present marked immune dysfunction due to age-related senescence of both innate immunity and adaptive immunity, which contribute to viral infections (21); thus, healthcare workers' vigilance is critical for patient selection. Although there are studies suggesting that comorbidities affect LOS (22), in our study, this could not be proven.

TSS refers to a scoring system that accurately evaluates lung involvement and is estimated by at least two well-trained and experienced radiologists to assess the severity of COVID-19 in CT scans (23). Studies suggest the use of chest CT to facilitate diagnosis in patients with a high rate of suspicion and a negative PCR (24, 25); nevertheless, in our study, CT was used in known cases. According to a meta-analysis by Böger B et al., the sensitivity of a CT scan might reach up to 97.2% (26). In addition, studies investigating patients with severe clinical presentation who require hospitalization support that the sensitivity of CT is higher than that of RT-PCR (7, 15). A higher TSS in the CT scan in suspicious cases and the absence of a positive RT-PCR probably indicate COVID-19. Therefore, it is suggested to repeat the RT-PCR testing (27, 28).

In the present study, we found that a higher TSS is independently correlated with longer hospital stay, despite the presence or absence of comorbidities, even the most common comorbidities such as diabetes mellitus type 2 (29). We have noticed a high TSS in cases with no comorbidity, indicating that disease severity impedes recovery. This might indicate that there is a genetic background predisposing to severe disease. Diverse susceptibility to severe disease might evolve genetic variation and associated polymorphisms. Several studies attempt to clarify this field, which is still under investigation (30).

A limited number of scientific studies have appraised the prognostic factors of the LOS in patients with COVID-19. Most studies have identified age as an aggravating factor associated with both severe disease and death (31, 32). A cohort study by Hua Zheng et al. has shown that age significantly lengthens the hospital stay in a population of 1,792 patients (32). Especially after the age of 40 years, individuals are prone to prolonged hospitalizations that might reach up to a month, or more in the elderly. Furthermore, Wu et al. considered age as a factor of severity and disease progression to ARDS (31), while Grasseli et al. have shown that older individuals are more likely to require ICU hospitalization (33). To our knowledge, there are no studies that investigate how the TSS affects LOS, pointing to the significance of our results. Due to the emerging conditions that COVID-19 has established, the vast majority of studies focus primarily on the therapeutic approach and innovative protocol development. However, a complete and detailed initial assessment of disease severity using computed tomography, as a tool used in the first 24 h after hospitalization, in combination with other factors can help determine the most appropriate type of treatment and time of treatment initiation.

The literature shows that cardiovascular disease impacts most of the comorbid conditions; in particular, patients with diabetes mellitus type 2 and hypertension exhibit substantial risk for severe disease (34). Despite that no significant association between type 2 diabetes mellitus and LOS has been advocated in our study, studies prove that diabetes increases inpatient time (29). Regarding hypertension, data support that it predisposes to severe disease, although association with LOS has not been established yet (35). In the present study, we found that these comorbidities are not independent predictors of prolonged hospitalization.

The main limitation of our study is its retrospective design. As a result, there are missing details from demographic data such as body mass index. Our results cannot be extrapolated to the general population as the results concern only severely ill patients and the study was a single-center study.

In conclusion, this study was performed in a large cohort of hospitalized patients affected by COVID-19 pneumonia to determine the characteristics of the LOS. By investigating all the clinical and laboratory admission factors as well as the imaging of each patient, we conclude that the age and total severity score of the CT of admission can predict the duration of hospitalization. Furthermore, the TSS is an index that has not been sufficiently utilized by clinicians, although it is useful in disease severity classification and further patient management. To the best of our knowledge, this is the first study to evaluate the contribution of the TSS to the LOS in COVID-19 pneumonia. Previous studies have identified age as a main contributor to LOS, and studies have shown that other comorbidities also play an important role in the LOS (29, 36). There are also data in the literature relating the TSS with the mortality rate or rate of ICU admission in COVID-19 patients (37, 38). However, this is a novel study suggesting that a higher TSS at the initial admission is related to longer hospitalizations. We have shown that CT scan findings can predict the inpatient duration along with patients' age. This result was not affected by the presence or absence of other comorbidities.

This is the first study to use the TSS as a predicting factor in patients with COVID-19 pneumonia. The results of our study can be used by healthcare professionals to maintain a high suspicion during admission and to escalate a more efficient and aggressive therapeutic approach earlier during hospitalization.

## Data availability statement

The raw data supporting the conclusions of this article will be made available by the authors, without undue reservation.

## Author contributions

AN, MK, TG, KM, and BN participated in the design and conception of the study, actively participated in subjects' enrollment, and analyzed and interpreted the data. AN and MK collected and recorded the data. EM and CM participated in the radiological assessment. AN, MK, and BN edited the final manuscript, corrected, and approved the final manuscript. All authors read, approved the final version of the manuscript, and agreed to submit it for publication.
